# Telomere Length as a Biomarker for Race-Related Health Disparities

**DOI:** 10.3390/genes12010078

**Published:** 2021-01-09

**Authors:** Vaithinathan Selvaraju, Megan Phillips, Anna Fouty, Jeganathan Ramesh Babu, Thangiah Geetha

**Affiliations:** 1 Department of Nutrition, Dietetics, and Hospitality Management, Auburn University, Auburn, AL 36849, USA; vzs0041@auburn.edu (V.S.); phillml@auburn.edu (M.P.); adf0023@auburn.edu (A.F.); jeganrb@auburn.edu (J.R.B.); 2 Boshell Metabolic Diseases and Diabetes Program, Auburn University, Auburn, AL 36849, USA

**Keywords:** telomere length, race, blood pressure, health disparities

## Abstract

Disparities between the races have been well documented in health and disease in the USA. Recent studies show that telomere length, a marker of aging, is associated with obesity and obesity-related diseases, such as heart disease and diabetes. The current study aimed to evaluate the connection between telomere length ratio, blood pressure, and childhood obesity. The telomere length ratio was measured in 127 children from both European American (EA) and African American (AA) children, aged 6–10 years old. AA children had a significantly high relative telomere to the single copy gene (T/S) ratio compared to EA children. There was no significant difference in the T/S ratio between normal weight (NW) and overweight/obese (OW/OB) groups of either race. Blood pressure was significantly elevated in AA children with respect to EA children. Hierarchical regression analysis adjusted for race, gender, and age expressed a significant relationship between the T/S ratio and diastolic pressure. Low T/S ratio participants showed a significant increase in systolic pressure, while a high T/S ratio group showed an increase in diastolic pressure and heart rate of AA children. In conclusion, our findings show that AA children have high T/S ratio compared to EA children. The high T/S ratio is negatively associated with diastolic pressure.

## 1. Introduction

Telomeres are present at the end of the chromosomes as shielding caps and protect the degradation targeted by the chromosome’s cellular DNA damage. Telomeres consist of a repeated number of tandem sequences, TTAGGG, incorporated with a protein complex [1]. Telomere shows a substantial variation among individuals from birth and decreases during aging. 

Obesity is a chronic disease in children and adults, developed in recent decades, and increases comorbidities in adulthood [2]. Worldwide obesity prevalence increased rapidly due to its high impact among children and adults [3]. Hence, it is imperative to treat young children to avoid obesity-related difficulties during their adulthood. The literature reveals that the correlation between obesity and telomere length have shown ambiguous results in adults. Some reports indicate a direct or inverse relationship between obesity and telomere length [4,5,6,7], but it differs in children. There is no significant difference in the telomere length reported between normal weight obese Caucasian children, but the obese adults had a shorter telomere length than the normal weight adults [8]. Very few studies discussed the correlation between telomere length and obesity due to lifestyle changes, suggesting that telomere length is either protected or extended while maintaining or reducing weight [4,5,9]. Even though overall telomere length is shorter in the obese state [10], studies conducted in older adults did not show any relationship between telomere length and obesity [5,7]. All individuals vary in telomere length with its reflection at birth [11], and the difference is observed throughout life [12,13]. Oxidative stress is a process that increases the shortening of telomeres during each cell division in somatic cells [14,15]. Inflammation is an additional factor responsible for telomere reduction in leukocytes by increasing hematopoietic stem cells [16]. Obesity is the foremost independent risk factor for increasing aging and it is associated with metabolic diseases, such as hypertension, diabetes, cardiovascular disease, and cancer [17,18,19,20,21]. Even though the causal directions of these relationships persist to be defined [22,23], shorter telomeres are a well-established biomarker for various age-related conditions [24]. 

Recent studies have shown that telomere length, a marker of cellular aging, is used in studies of race-based health disparities and it is sensitive to effects of social stress [25,26,27,28]. Notably, African Americans have high levels of poor health, and it is expected for African Americans to have shorter telomeres than European Americans (EAs). Interestingly, recent findings show that African Americans (AAs) have longer telomere length in contrast to the expectations [29,30,31,32,33]. 

In industrialization, an age-related rise in pulse pressure is used as a phenotype of biological aging of the vasculature. In almost 95% of the hypertensive cases, hypertension is unknown [34]. Telomere attrition has been linked through hypertension and, during aging, it has been found in cells such as vascular endothelial cells, smooth muscle cells, and cardiomyocytes [35]. Therefore, in the current study, we examined the relationship between telomere length and blood pressure among EA and AA children. The relative telomere length to a single copy gene (T/S ratio) was calculated by comparing the telomeric DNA (T) level with the single copy (S) hemoglobin subunit γ one gene in salivary genomic DNA by quantitative real-time PCR. First, we tested whether the mean T/S ratio differed across the overall participants’ races or weight category and low and high T/S ratio groups. Second, we tested the relationship between blood pressure and heart rate with low and high T/S ratios.

## 2. Materials and Methods

### 2.1. Study Subjects

The Auburn University Institutional Review Board for the Protection of Human Subjects in Research approved this study. The written consent was obtained from the participants and their parents before sample collection. By distributing the study flyer in the Lee and Macon counties in Alabama, participants were recruited from schools, after-school programs, through friends, and via participant referrals. Participants were recruited between ages 6–10 years, and a phone survey was conducted before recruiting to exclude the participants with health conditions such as diabetes and cardiovascular disease. 

### 2.2. Anthropometric Measurements and Saliva Collection

The participants’ body weight and height were measured, as described in our previous study [36]. The body mass index (BMI) percentile range was calculated according to the Centers for Diseases Control and Prevention (CDC), and the participants were grouped into normal weight and overweight/obese [37]. Saliva collection and isolation of salivary DNA was performed, as previously discussed [36]. The DNA isolated was used to measure the telomere length.

### 2.3. Blood Pressure Measurement

Measurement of blood pressure (BP) is known as an integral part of clinical examinations. The “gold standard” method was used to record the BP in participants as auscultatory, with an aneroid non-mercury manometer connected to a suitable cuff for the children. The systolic and diastolic pressure, along with heart rate, was recorded.

### 2.4. Telomere Length Measurement

The telomere length was measured as previously described in Cawthon et al. (2002), using a QuantStudio 3 real-time polymerase chain reaction (PCR) system [38]. The qPCR reaction mixture was performed with a volume of 20 µl using the following reagents. Assays were performed using 96-well plates (Thermo Fisher Scientific, Waltham, MA, USA) and the qPCR mix contained SYBR green PCR master mix (which included ROX passive dye), telomere primers (Tel 1, 5’-GGT TTT TGA GGG TGA GGG TGA GGG TGA GGG TGA GGG T-3’ and Tel 2, 5’-TCC CGA CTA TCC CTA TCC CTA TCC CTA TCC CTA TCC CTA-3’) (Integrated DNA Technologies, Inc., Coralville, IA, USA), and RNase free water to adjust the reaction volume. With this qPCR mix, a template DNA was added to perform the PCR. Simultaneously, single-copy gene qPCR was performed by preparing the qPCR mix of SYBR green PCR master mix with ROX passive dye, single copy number gene primers (hbg 1, 5’-GCT TCT GAC ACA ACT GTG TTC ACT AGC -3’ and hbg 2, 5’-CAC CAA CTT CAT CCA CGT TCA CC-3’) (Integrated DNA Technologies, Inc., Coralville, IA, USA), and RNase free water, along with template DNA. The telomere PCR and single-copy gene PCR was performed in separate 96-well plates matching the same well position of telomere PCR in the first plate and single-copy gene PCR on the second plate. The plates were prepared with six-point reference HeLa DNA (New England BioLabs, Ipswich, MA, USA) from the highest concentration of 150 ng/mL three-fold dilution in duplicates to make a standard curve. There were two negative control wells and four positive control wells (equivalent concentration of sample DNA template) on every plate. Plates were sealed with transparent optical adhesive covers (Thermo Fisher Scientific, Waltham, MA, USA) and loaded in the QuantStudio 3 real-time PCR system. The reaction was carried out using the following cycling profile of initiation for 10 min at 95 °C, followed by 40 cycles of denaturation at 95 °C for 20 s, annealing at 52 °C for 20 s and extension at 72 °C for 45 s with signal acquisition; and then a melt curve was performed. 

For each run of qPCR, amplification curves and melt curves were inspected to evaluate the sample and qPCR run quality, along with the absence of amplification in negative controls. Before calculating the T/S values, a coefficient variation of an inter and intra assay was examined. The relative measure of telomere length and T/S ratio in each sample was calculated in a separate excel sheet using the following formula:

ΔCt = Ct_Tel_ − Ct_hbg_

ΔCt of control = Ct_Tel_ − Ct_control_

ΔΔCt = ΔCt − ΔCt of control

Relative T/S = 2^−^^ΔΔCt^

The low and high T/S ratio groups were separated based on the median T/S ratio. The low T/S ratio group denotes that the T/S ratio values were less than the median. On the other hand, the participant’s T/S ratio was higher than the median, named a high T/S ratio group.

### 2.5. Statistical Analysis

All the data in the graphical representation was expressed as mean ± SEM, *p* < 0.05 considered as significant. The descriptive and hierarchical regression analyses were performed using the Statistical Package for Social Sciences (SPSS, version 25, IBM, Armonk, NY, USA). We used hierarchical regression analysis, introducing covariates such as race, age, and gender to predict the dependent variables. A two tailed Students t-test was used to calculate the mean difference between the two groups. The graphs were prepared using Graphpad Prism version 8 (San Diego, CA, USA). The proportion graphs were prepared using the number of participants in each category of study. The statistically significant difference in proportion graphs was calculated using the online MedCalc software website (Ostend, Belgium). 

## 3. Results

The anthropometric measurements, telomere length ratio, and blood pressure for low and high T/S ratio groups are listed in Table 1 from the 127 participants, aged 6–10 years. There was no significant difference observed between the low and high T/S ratio of age, height, weight, and BMI. The T/S ratio did not show a significant difference between EA and AA children, normal weight (NW) and overweight/obese (OW/OB) groups. Systolic, diastolic pressure and pulse were among the low and high T/S ratio groups not showing significant changes. The mean T/S ratio of all participants was 1.06 ± 0.04, with a median of 1.066. The mean T/S ratio of EA participants was 0.907 ± 0.04 (median 0.805) and AA participants was 1.23 ± 0.07 (median 1.216). The mean T/S ratios of NW and OW/OB participants were 1.07 ± 0.04 and 1.04 ± 0.07, respectively.

**Figure 1:**
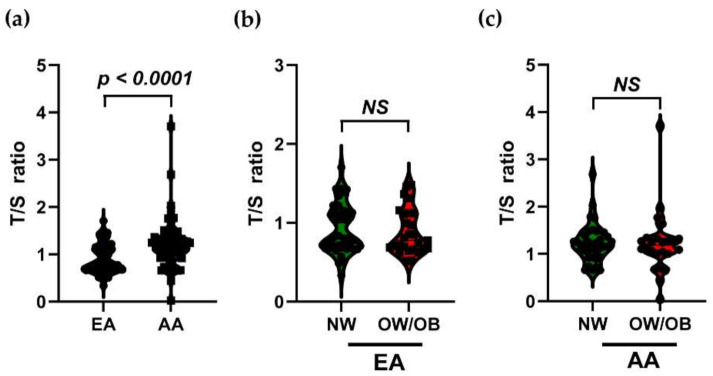
The violin plot with data points shows the distribution of the T/S ratio in EA and AA children. (**a**) The mean T/S ratio is compared between EA and EA children. The T/S ratio of NW and OW/OB children is compared in EA (**b**) and AA children (**c**). Values are expressed as mean ± SEM. European American (EA); African American (AA); normal weight (NW); overweight/obese (OW/OB); telomere length/ single copy gene (T/S ratio); Not significant (NS).

To determine the difference in the telomere length ratio between races, the calculated T/S ratio was analyzed by the t-test. AA participants showed a significantly (*p <* 0.0001) higher T/S ratio compared to EA participants (Figure 1a). The telomere length ratios among races for NW and OW/OB groups were also compared. The NW and OW/OB participants did not show any significant differences in either EA participants (NW-0.922 ± 0.05; OW/OB-0.883 ± 0.05) or AA participants (NW-1.235 ± 0.07; OW/OB-1.22 ± 0.14) (Figure 1b,c).

**Figure 2:**
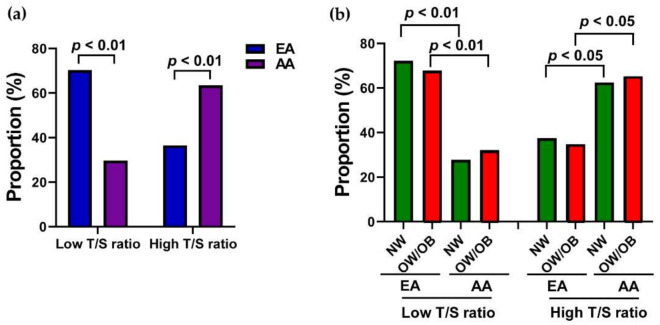
Proportion of participants with low and high T/S ratio groups. (**a**) The proportion difference participants with low and high T/S ratios in EA and AA children. (**b**) The proportion difference among NW and OW/OB children of EA and AA participants with low and high T/S ratios.

Based on the median T/S ratio, the study group was divided into two: low and high T/S ratios. The proportion of the low T/S ratio participants was significantly higher in the EA group (70.31%) than the AA group (29.69%). On the contrary, a greater proportion of high T/S ratio participants were found in the AA group (63.49%) in comparison to the EA group (36.51%), as shown in Figure 2a. The proportion of participants with low T/S ratios in both NW and OW/OB groups of the AA participants decreased significantly (*p <* 0.01) compared to EA participants of NW and OW/OB groups. However, the proportion of high T/S ratios in both NW and OW/OB groups of AA participants significantly (*p <* 0.05) increased in comparison to corresponding EA participants (Figure 2b). This result suggests that the T/S ratio in AA participants significantly increased compared to EA participants, but there was no difference between the NW and OW/OB groups with EA or AA children.

**Figure 3:**
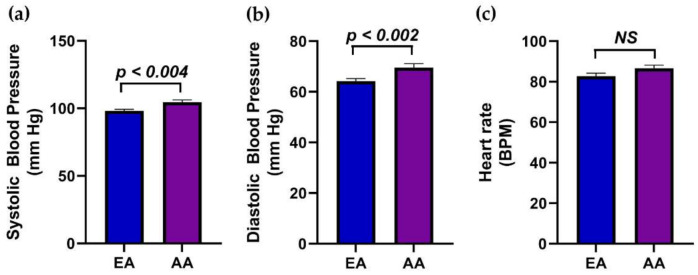
The blood pressure of EA and AA participants. The difference in the (**a**) systolic pressure, (**b**) diastolic pressure, and (**c**) heart rate between EA and AA children. Values are expressed as mean ± SEM.

**Figure 4:**
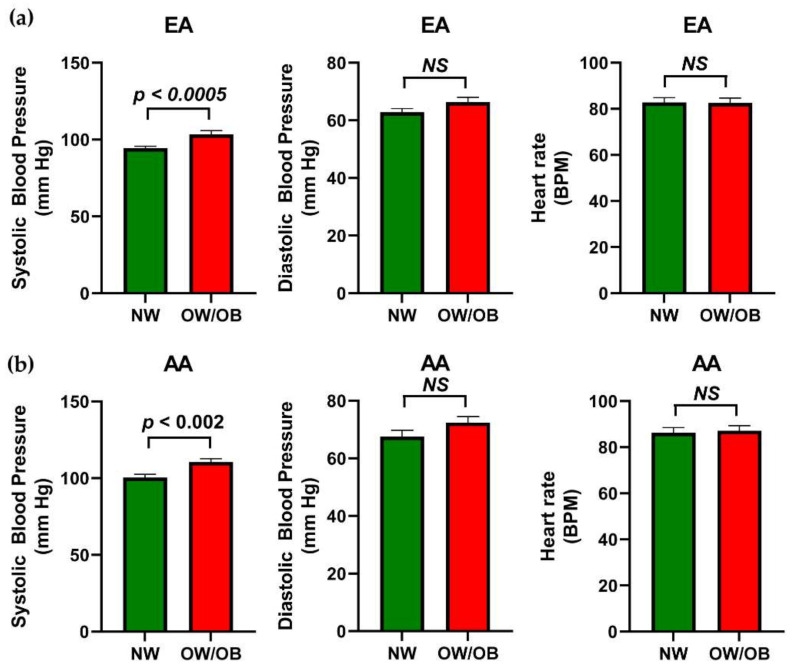
Comparison of blood pressure in NW and OW/OB children of EA and AA participants. The bar graphs shows the differences in the blood pressure in the NW and OW/OB groups of (**a**) EA participants and (**b**) AA participants. Values are expressed as mean ± SEM.

Next, the difference in the circulatory pressure between race, NW, and OW/OB participants was determined. The systolic blood pressure (*p <* 0.004) and diastolic pressure (*p <* 0.002) among AA participants significantly increased compared to the EA group (Figure 3a,b). However, there was no difference observed between AA and EA participants’ pulse rates (Figure 3c). The OW/OB participants of both the EA and AA groups had significantly higher systolic blood pressure compared to NW participants. However, the diastolic pressure and pulse rate among NW and OW/OB groups of EA and AA participants did not show significant changes (Figure 4a,b). This suggests that the systolic and diastolic pressure increased in the AA participants compared to EA participants but only the systolic pressure in OW/OB was greater than the NW group of EA and AA participants.

**Figure 5:**
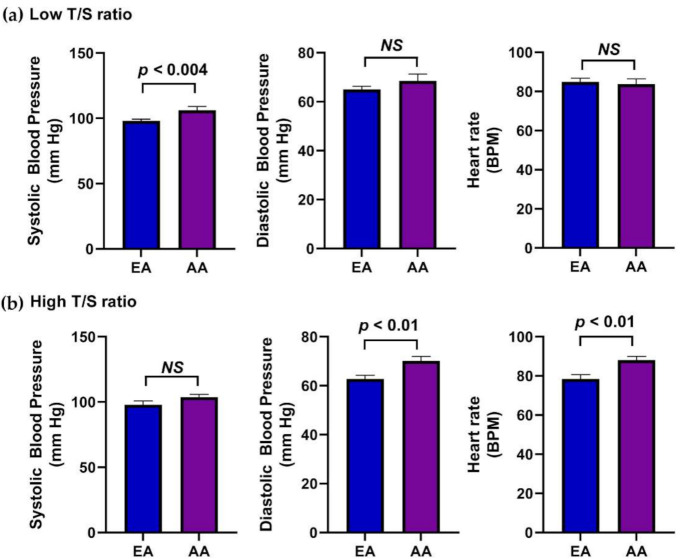
Comparison of blood pressure in EA and AA children with low and high T/S ratios. Bar graphs shows the differences in blood pressure in EA and AA participants with (**a**) the low T/S ratio and (**b**) the high T/S ratio. Values are expressed as mean ± SEM.

The differences in the blood pressure and heart rate of the low and high T/S ratio participants were determined between EA and AA groups. The systolic pressure was significantly increased (*p <* 0.004) in AA participants compared to EA participants with low T/S ratios. However, there was no difference in diastolic pressure and heart rate with low T/S ratios (Figure 5a). AA participants with a high T/S ratio observed significantly increased diastolic pressure (*p <* 0.01) and heart rate (*p <* 0.01) compared to the EA group. Nevertheless, systolic blood pressure did not significantly change in high T/S ratio participants (Figure 5b). This explains that the AA children with low T/S ratios had higher systolic pressure and those with high T/S ratios had higher diastolic pressure and heart rate compared to EA children.

The relationship between the T/S ratio and circulatory pressure for unadjusted and adjusted hierarchical regression was analyzed. Race, gender, and age of the participants were entered to adjust for covariates. Both adjusted and unadjusted hierarchical regression was performed with all participants for low and high T/S ratio groups between T/S ratios, systolic, diastolic pressure, and heart rate. Even though systolic pressure showed positive and diastolic pressure, it showed a negative correlation with the T/S ratio. There was no significant difference observed in the β-coefficient of unadjusted regression analysis. After adjusting for race, gender, and age, diastolic pressure showed a significant negative correlation (β = −0.327; *p* < 0.049) with high T/S ratio participants (Table 2). This suggests that children with high T/S ratios are negatively correlated with diastolic pressure.

## 4. Discussion

The current study includes 68 EA and 59 AA (total 127) children with an age range of 6–10 years. The study population was divided into low and high T/S ratios based on the median. We did not observe differences in the T/S ratio according to age, height, weight, or weight category. The results agree with previous studies in children and adult populations [39,40]. This may be due to the narrow range of ages and sample sizes used in the study. The mean T/S ratio in the AA children increased compared to the EA children. Similar results were found by Rewak et al., with significantly longer leucocyte telomere length (LTL) in Blacks than Whites at birth and adulthood [41]. Several studies showed longer LTL on average in AA participants [29,30,31,32,33]. In adulthood, shorter leukocyte telomeres are correlated with BMI in women [42] and waist-hip ratios in both genders [13]. However, previous studies on childhood obesity and telomere length showed an inconclusive result. The NW and OW/OB children in both EA and AA participants did not show any significant changes in the mean T/S ratio. These results are in correlation with Zannolli et al., where the leukocyte telomere length did not show any difference between obese and non-obese Italian children [8]. The participants were separated based on the T/S ratio’s median into low and high T/S ratio groups. In the high T/S ratio group, the proportion of the AA children was more compared to EA children, and the proportion of participants of NW and OW/OB children in the AA group showed higher numbers. This result clearly correlates with the previous results published by Rewak et al. on the childhood telomere length [41]. 

Systolic and diastolic pressure was significantly increased in AA children compared to EA children. Globalization increases systolic blood pressure throughout life [43,44]. Diastolic blood pressure is also increased in early life, but it tends to decrease in aged persons [45]. Arterial aging has predominantly increased the stiffness of central elastic arteries, which is the factor that regulates pulse pressure. The factors that increase the biological aging of vessel formation, including essential hypertension [46], other diseases, such as diabetes [47], and higher amounts of salt intake [48], independently increase the arterial stiffness. Connecting all these processes proposes that aortic blood pressure might show a phenotype of aging and is one of the major risk factors for cardiovascular disease [49,50,51]. 

The systolic pressure of the OW/OB children of both EA and AA races was significantly increased than in NW children. The overall T/S ratio was high in AA participants, and the diastolic blood pressure and heart rate also increased in the same participant group. Our results are consistent with earlier cross-sectional findings of race/ethnicity differences in telomere length at different age groups. In particular, Rewak et al. showed a longer telomere length in the AA population [41]. The study conducted with NHANES data suggests that cardiovascular health is associated with smaller leucocyte telomere lengths and race/ethnicity [52]. Numerous studies have consistently reported that more prevalence of hypertension in AA participants than EA participants is the primary reason for increased cardiovascular disease occurrence in the AA community [53]. Based on the median of the T/S ratio, significantly increased diastolic pressure and heart rate was observed in the high T/S ratio of AA participants. The shorter telomere lengths are correlated with hypertension in adults [54]. We found that systolic pressure was only significantly increased in AA children with a low T/S ratio. Additionally, our results suggest that the characteristic is only a significant analyst of telomere length in adulthood and not in childhood. Aydos and Tukun (2007) describe no correlation between systolic pressure, diastolic pressure, BMI, and telomere length [55]. However, in this study, the hierarchical regression analysis, after adjusting with covariates such as race, gender, and age, indicates that children with high T/S ratios are negatively correlated with diastolic pressure. 

Some limitations need to be acknowledged. We used a real-time PCR method to measure the telomere length ratio with higher assay variability in general than the traditional telomere restriction fragment method [56,57]. The plate-to-plate variability effect was minimized using the corresponding well layout of the telomere primer and single-copy gene [9]. The results should also be considered preliminary due to the small sample size in the study. 

## 5. Conclusions

In this study, we explored the connection between telomere length ratio, blood pressure and childhood obesity. Our results demonstrated that AA children have a greater mean T/S ratio and systolic and diastolic pressure compared to EA children. The OW/OB children of both EA and AA groups have higher systolic pressure compared to NW children. AA children with low T/S ratios have increased systolic pressure and children with high T/S ratios have increased diastolic pressure and heart rate than EA children. The children with high T/S ratio are negatively correlated with diastolic pressure. This study validates the use of non-invasive salivary measurement of telomere length and addresses the gap between the T/S ratio and blood pressure among children. 

## Figures and Tables

**Table 1:** General characteristics of the study population.

	Low T/S Ratio (64)	High T/S Ratio (63)	*p* Value
Age (year)	8.40 ± 0.19	8.19 ± 0.18	*p* < 0.427
Height (cm)	133.01 ± 1.42	130.89 ± 1.45	*p* < 0.297
Weight (kg)	32.39 ± 1.34	32.07 ± 1.42	*p* < 0.868
BMI (kg/m^2^)	17.98 ± 0.41	18.21 ± 0.45	*p* < 0.696
T/S Ratio			
All	0.739 ± 0.02	1.38 ± 0.05	*p* < 0.0001
EA	0.724 ± 0.02	1.26 ± 0.04	*p* < 0.0001
AA	0.776 ± 0.06	1.44 ± 0.08	*p* < 0.0001
NW	0.750 ± 0.03	1.35 ± 0.05	*p* < 0.0001
OW/OB	0.726 ± 0.04	1.43 ± 0.11	*p* < 0.0001
Blood Pressure			
Systolic Pressure (mmHg)	100.52 ± 1.31	101.61 ± 1.72	*p* < 0.610
Diastolic Pressure (mmHg)	66.05 ± 1.24	67.38 ± 1.40	*p* < 0.477
Heart rate (BPM)	84.52 ± 1.58	84.51 ± 1.57	*p* < 0.997

European American (EA); African American (AA); normal weight (NW); overweight/obese (OW/OB); telomere length/ single copy gene (T/S ratio); values are expressed as mean ± SEM.

**Table 2:** The relationship between T/S ratio and blood pressure by hierarchical regression analysis (Adjusted for race, gender, and age).

	Unadjusted						Adjusted					
Parameters	B	SE	Β-Coefficient	95% Confidence Interval for b		*p*-Value	B	SE	Β-Coefficient	95% Confidence Interval for b		*p*-Value
				Lower Bound	Upper Bound					Lower Bound	Upper Bound	
All participants												
Systolic Pressure	0.005	0.004	0.136	−0.003	0.014	0.242	0.003	0.004	0.079	−0.006	0.011	0.497
Diastolic Pressure	−0.005	0.005	−0.113	−0.015	0.005	0.333	−0.007	0.005	−0.162	−0.016	0.002	0.143
Pulse	0.000	0.003	0.012	−0.006	0.007	0.891	−0.002	0.003	−0.057	−0.008	0.004	0.508
Low T/S ratio participants												
Systolic Pressure	0.000	0.003	0.017	−0.006	0.006	0.923	−0.001	0.004	−0.051	−0.008	0.006	0.800
Diastolic Pressure	−0.003	0.003	−0.135	−0.009	0.004	0.444	−0.001	0.003	−0.077	−0.008	0.005	0.679
Pulse	−0.001	0.002	−0.093	−0.005	0.002	0.474	−0.001	0.002	−0.090	−0.005	0.003	0.495
High T/S ratio participants												
Systolic Pressure	0.007	0.005	0.251	−0.002	0.017	0.122	0.006	0.005	0.205	−0.004	0.016	0.228
Diastolic Pressure	−0.010	0.006	−0.259	−0.021	0.002	0.109	−0.012	0.006	−0.327	−0.024	0.000	**0.049**
Pulse	0.003	0.004	0.085	−0.006	0.011	0.508	−0.001	0.005	−0.034	−0.010	0.008	0.809

Unstandardized coefficient (B); standard error (SE). The bold number shows the significant.

## Data Availability

The data presented in this study is available on request from the corresponding author.

## References

[1] Blackburn E.H. (1991). Structure and function of telomeres. Nature.

[2] Biro F.M., Wien M. (2010). Childhood obesity and adult morbidities. Am. J. Clin. Nutr..

[3] de Onis M., Blossner M., Borghi E. (2010). Global prevalence and trends of overweight and obesity among preschool children. Am. J. Clin. Nutr..

[4] Cui Y., Gao Y.T., Cai Q., Qu S., Cai H., Li H.L., Wu J., Ji B.T., Yang G., Chow W.H. (2013). Associations of leukocyte telomere length with body anthropometric indices and weight change in Chinese women. Obesity.

[5] Garcia-Calzon S., Gea A., Razquin C., Corella D., Lamuela-Raventos R.M., Martinez J.A., Martinez-Gonzalez M.A., Zalba G., Marti A. (2014). Longitudinal association of telomere length and obesity indices in an intervention study with a Mediterranean diet: The PREDIMED-NAVARRA trial. Int. J. Obes..

[6] Lee M., Martin H., Firpo M.A., Demerath E.W. (2011). Inverse association between adiposity and telomere length: The Fels Longitudinal Study. Am. J. Hum. Biol..

[7] Njajou O.T., Cawthon R.M., Blackburn E.H., Harris T.B., Li R., Sanders J.L., Newman A.B., Nalls M., Cummings S.R., Hsueh W.C. (2012). Shorter telomeres are associated with obesity and weight gain in the elderly. Int. J. Obes..

[8] Zannolli R., Mohn A., Buoni S., Pietrobelli A., Messina M., Chiarelli F., Miracco C. (2008). Telomere length and obesity. Acta Paediatr..

[9] O’Callaghan N.J., Fenech M. (2011). A quantitative PCR method for measuring absolute telomere length. Biol. Proced. Online.

[10] Mundstock E., Sarria E.E., Zatti H., Louzada F.M., Grun L.K., Jones M.H., Guma F.T., Mazzola J., Epifanio M., Stein R.T. (2015). Effect of obesity on telomere length: Systematic review and meta-analysis. Obesity.

[11] Okuda K., Bardeguez A., Gardner J.P., Rodriguez P., Ganesh V., Kimura M., Skurnick J., Awad G., Aviv A. (2002). Telomere length in the newborn. Pediatr. Res..

[12] Chen W., Gardner J.P., Kimura M., Brimacombe M., Cao X., Srinivasan S.R., Berenson G.S., Aviv A. (2009). Leukocyte telomere length is associated with HDL cholesterol levels: The Bogalusa heart study. Atherosclerosis.

[13] Farzaneh-Far R., Lin J., Epel E., Lapham K., Blackburn E., Whooley M.A. (2010). Telomere length trajectory and its determinants in persons with coronary artery disease: Longitudinal findings from the heart and soul study. PLoS ONE.

[14] Kurz D.J., Decary S., Hong Y., Trivier E., Akhmedov A., Erusalimsky J.D. (2004). Chronic oxidative stress compromises telomere integrity and accelerates the onset of senescence in human endothelial cells. J. Cell Sci..

[15] von Zglinicki T. (2002). Oxidative stress shortens telomeres. Trends Biochem. Sci..

[16] Aviv A., Valdes A.M., Spector T.D. (2006). Human telomere biology: Pitfalls of moving from the laboratory to epidemiology. Int. J. Epidemiol..

[17] Boles A., Kandimalla R., Reddy P.H. (2017). Dynamics of diabetes and obesity: Epidemiological perspective. Biochim. Biophys. Acta Mol. Basis Dis..

[18] Csige I., Ujvarosy D., Szabo Z., Lorincz I., Paragh G., Harangi M., Somodi S. (2018). The Impact of Obesity on the Cardiovascular System. J. Diabetes Res..

[19] Leung M.Y., Carlsson N.P., Colditz G.A., Chang S.H. (2017). The Burden of Obesity on Diabetes in the United States: Medical Expenditure Panel Survey, 2008 to 2012. Value Health.

[20] Seravalle G., Grassi G. (2017). Obesity and hypertension. Pharmacol. Res..

[21] Van Gaal L.F., Mertens I.L., De Block C.E. (2006). Mechanisms linking obesity with cardiovascular disease. Nature.

[22] Aviv A. (2009). Leukocyte telomere length, hypertension, and atherosclerosis: Are there potential mechanistic explanations?. Hypertension.

[23] Testa R., Ceriello A. (2007). Pathogenetic loop between diabetes and cell senescence. Diabetes Care.

[24] Armanios M., Blackburn E.H. (2012). The telomere syndromes. Nat. Rev. Genet..

[25] Cherkas L.F., Aviv A., Valdes A.M., Hunkin J.L., Gardner J.P., Surdulescu G.L., Kimura M., Spector T.D. (2006). The effects of social status on biological aging as measured by white-blood-cell telomere length. Aging Cell.

[26] Epel E.S., Blackburn E.H., Lin J., Dhabhar F.S., Adler N.E., Morrow J.D., Cawthon R.M. (2004). Accelerated telomere shortening in response to life stress. Proc. Natl. Acad. Sci. USA.

[27] Epel E.S., Lin J., Wilhelm F.H., Wolkowitz O.M., Cawthon R., Adler N.E., Dolbier C., Mendes W.B., Blackburn E.H. (2006). Cell aging in relation to stress arousal and cardiovascular disease risk factors. Psychoneuroendocrinology.

[28] Simon N.M., Smoller J.W., McNamara K.L., Maser R.S., Zalta A.K., Pollack M.H., Nierenberg A.A., Fava M., Wong K.K. (2006). Telomere shortening and mood disorders: Preliminary support for a chronic stress model of accelerated aging. Biol. Psychiatry.

[29] Chen W., Kimura M., Kim S., Cao X., Srinivasan S.R., Berenson G.S., Kark J.D., Aviv A. (2011). Longitudinal versus cross-sectional evaluations of leukocyte telomere length dynamics: Age-dependent telomere shortening is the rule. J. Gerontol. A Biol. Sci. Med. Sci..

[30] Diaz V.A., Mainous A.G., Player M.S., Everett C.J. (2010). Telomere length and adiposity in a racially diverse sample. Int. J. Obes..

[31] Fitzpatrick A.L., Kronmal R.A., Kimura M., Gardner J.P., Psaty B.M., Jenny N.S., Tracy R.P., Hardikar S., Aviv A. (2011). Leukocyte telomere length and mortality in the Cardiovascular Health Study. J. Gerontol. A Biol. Sci. Med. Sci..

[32] Hunt S.C., Chen W., Gardner J.P., Kimura M., Srinivasan S.R., Eckfeldt J.H., Berenson G.S., Aviv A. (2008). Leukocyte telomeres are longer in African Americans than in whites: The National Heart, Lung, and Blood Institute Family Heart Study and the Bogalusa Heart Study. Aging Cell.

[33] Zhu H., Wang X., Gutin B., Davis C.L., Keeton D., Thomas J., Stallmann-Jorgensen I., Mooken G., Bundy V., Snieder H. (2011). Leukocyte telomere length in healthy Caucasian and African-American adolescents: Relationships with race, sex, adiposity, adipokines, and physical activity. J. Pediatr..

[34] Cowley A.W. (2006). The genetic dissection of essential hypertension. Nat. Rev. Genet..

[35] Edo M.D., Andres V. (2005). Aging, telomeres, and atherosclerosis. Cardiovasc. Res..

[36] Venkatapoorna C.M.K., Ayine P., Parra E.P., Koenigs T., Phillips M., Babu J.R., Sandey M., Geetha T. (2019). Association of Salivary Amylase (AMY1) Gene Copy Number with Obesity in Alabama Elementary School Children. Nutrients.

[37] Kuczmarski R.J., Ogden C.L., Guo S.S., Grummer-Strawn L.M., Flegal K.M., Mei Z., Wei R., Curtin L.R., Roche A.F., Johnson C.L. (2002). 2000 CDC Growth Charts for the United States: Methods and Development.

[38] Cawthon R.M. (2002). Telomere measurement by quantitative PCR. Nucleic Acids Res..

[39] Al-Attas O.S., Al-Daghri N., Bamakhramah A., Sabico S.S., McTernan P., Huang T.T. (2010). Telomere length in relation to insulin resistance, inflammation and obesity among Arab youth. Acta Paediatr..

[40] Buxton J.L., Walters R.G., Visvikis-Siest S., Meyre D., Froguel P., Blakemore A.I. (2011). Childhood obesity is associated with shorter leukocyte telomere length. J. Clin. Endocrinol. Metab..

[41] Rewak M., Buka S., Prescott J., De Vivo I., Loucks E.B., Kawachi I., Non A.L., Kubzansky L.D. (2014). Race-related health disparities and biological aging: Does rate of telomere shortening differ across blacks and whites?. Biol. Psychol..

[42] Nordfjall K., Svenson U., Norrback K.F., Adolfsson R., Lenner P., Roos G. (2009). The individual blood cell telomere attrition rate is telomere length dependent. PLoS Genet..

[43] Franklin S.S., Gustin W.t., Wong N.D., Larson M.G., Weber M.A., Kannel W.B., Levy D. (1997). Hemodynamic patterns of age-related changes in blood pressure. The Framingham Heart Study. Circulation.

[44] Whelton P.K., He J., Klag M.J., Swales J.D. (1994). Blood pressure in Westernized population. Textbook of Hypertension.

[45] Pinto E. (2007). Blood pressure and ageing. Postgrad. Med. J..

[46] Gribbin B., Pickering T.G., Sleight P. (1979). Arterial distensibility in normal and hypertensive man. Clin. Sci..

[47] Salomaa V., Riley W., Kark J.D., Nardo C., Folsom A.R. (1995). Non-insulin-dependent diabetes mellitus and fasting glucose and insulin concentrations are associated with arterial stiffness indexes. The ARIC Study. Atherosclerosis Risk in Communities Study. Circulation.

[48] Avolio A.P., Clyde K.M., Beard T.C., Cooke H.M., Ho K.K., O’Rourke M.F. (1986). Improved arterial distensibility in normotensive subjects on a low salt diet. Arteriosclerosis.

[49] Benetos A., Rudnichi A., Safar M., Guize L. (1998). Pulse pressure and cardiovascular mortality in normotensive and hypertensive subjects. Hypertension.

[50] Domanski M.J., Davis B.R., Pfeffer M.A., Kastantin M., Mitchell G.F. (1999). Isolated systolic hypertension: Prognostic information provided by pulse pressure. Hypertension.

[51] Verdecchia P., Schillaci G., Borgioni C., Ciucci A., Pede S., Porcellati C. (1998). Ambulatory pulse pressure: A potent predictor of total cardiovascular risk in hypertension. Hypertension.

[52] Gebreab S.Y., Manna Z.G., Khan R.J., Riestra P., Xu R., Davis S.K. (2017). Less than Ideal Cardiovascular Health Is Associated with Shorter Leukocyte Telomere Length: The National Health and Nutrition Examination Surveys, 1999–2002. J. Am. Heart Assoc..

[53] Lloyd-Jones D., Adams R.J., Brown T.M., Carnethon M., Dai S., De Simone G., Ferguson T.B., Ford E., Furie K., Gillespie C. (2010). Executive summary: Heart disease and stroke statistics—2010 update: A report from the American Heart Association. Circulation.

[54] Yang Z., Huang X., Jiang H., Zhang Y., Liu H., Qin C., Eisner G.M., Jose P.A., Rudolph L., Ju Z. (2009). Short telomeres and prognosis of hypertension in a chinese population. Hypertension.

[55] Aydos S.E., Tukun A. (2007). Does telomere length affect blood pressure?. Adv. Ther..

[56] Aviv A., Hunt S.C., Lin J., Cao X., Kimura M., Blackburn E. (2011). Impartial comparative analysis of measurement of leukocyte telomere length/DNA content by Southern blots and qPCR. Nucleic Acids Res..

[57] Kimura M., Stone R.C., Hunt S.C., Skurnick J., Lu X., Cao X., Harley C.B., Aviv A. (2010). Measurement of telomere length by the Southern blot analysis of terminal restriction fragment lengths. Nat. Protoc..

